# Cardiovascular Vertigo

**Published:** 1891-02-07

**Authors:** 


### MEDICINE.

### CARDIOVASCULAR VERTIGO.

This form of vertigo commonly occurs in association with symptoms of cerebral thrombosis. The vertigo of chronic nicotism, and that seen in gout and rheumatism, are, according to Grasset (*Sem. Méd.*) of the same nature. It is one of the first symptoms of commencing sclerosis of the arteries, and is caused by any temporary disturbance of the circulation in the vessels of the brain. Such attacks of vertigo occurring in elderly persons are often interpreted as "apoplexies," but being really rather functional than structural need not excite apprehension of immediately grave results, though indicating commencing degeneration and calling for careful supervision of the general habits of life.

